# Id4 Suppresses the Growth and Invasion of Colorectal Cancer HCT116 Cells through CK18-Related Inhibition of AKT and EMT Signaling

**DOI:** 10.1155/2021/6660486

**Published:** 2021-04-14

**Authors:** Hui-Jing Chen, Yue Yu, Yan-Xia Sun, Chuan-Zhong Huang, Jie-Yu Li, Fang Liu, Guo-Xiang Guo, Yun-Bin Ye

**Affiliations:** ^1^Laboratory of Immuno-Oncology, Fujian Cancer Hospital and Fujian Medical University Cancer Hospital, Fuzhou 350014, China; ^2^School of Basic Medical Sciences, Fujian Medical University, Fuzhou 350122, China; ^3^Fujian Provincial Key Laboratory of Translational Cancer Medicine, Fuzhou 350014, China

## Abstract

Id4 is one of the inhibitors of DNA-binding proteins (Id) and involved in the pathogenesis of numerous cancers. The specific mechanism underlying the Id4-mediated regulation of proliferation, invasion, and metastasis of colorectal cancer (CRC) cells is still largely unclear. In the present study, results showed CRC cells had a lower baseline Id4 expression than normal intestinal epithelial NCM460 cells. In order to explore the role of Id4 in the tumorigenicity, CRC HCT116 cells with stable Id4 expression were used, and results showed Id4 overexpression arrested the cell cycle at the G0/G1 phase, inhibited the cell proliferation and the colony formation, as well as suppressed the migration and invasion. In the in vivo model, Id4 overexpression inhibited the tumor growth and metastasis in the nude mice. Furthermore, Id4 overexpression upregulated the expression of proteins associated with cell proliferation, inhibited the PI3K/AKT pathway, and suppressed epithelial-mesenchymal transition (EMT) of HCT116 cells. Moreover, Id4 significantly decreased cytokeratin 18 (CK18) expression, but CK18 overexpression in Id4 expressing HCT116-Id4 cells rescued the activation of AKT, p-AKT, MMP2, MMP7, and E-cadherin. Collectively, our study indicated Id4 may inhibit CRC growth and metastasis through inhibiting the PI3K/AKT pathway in a CK18-dependent manner and suppressing EMT. Id4 may become a target for the treatment of CRC.

## 1. Introduction

Colorectal cancer (CRC) is one of the most common malignancies worldwide with more than 1 million new cases and 551 thousand CRC-related deaths in 2018 [1]. About 25% of CRC patients present with metastases at initial diagnosis [2]. The activation of oncogenes and inactivation of tumor suppressor genes have been shown to be related to the occurrence and development of many cancers, including CRC, but the molecular mechanisms underlying the pathogenesis and metastasis of CRC are still poorly understood.

Inhibitors of DNA-binding (also called inhibitors of cell differentiation, Id) belong to the family of classic basic helix-loop-helix (bHLH) transcription factors, but they lack a DNA-binding domain [3]. The Id proteins form a heterodimer with bHLH transcription factors, which covers their DNA-binding domain, and then inhibits its transcriptional activity. Id family members are overexpressed in some cancers such as CRC and have been shown not only to promote tumor growth, invasion, and metastasis but also to maintain the self-renewal of embryonic stem cells. Unlike other known Id family members, Id4 contains a highly conserved HLH motif in the dominant negative HLH (called dnHLH) proteins. The other three regions of Id4 protein are similar to those in other members of dnHLH protein family, but less conservative [4]. Previous studies have shown that Id4 expression is dysregulated in some human cancers, and Id4 may act as a tumor suppressor to inhibit cell proliferation and increase cell apoptosis in prostate cancer [5]. On the contrary, Id4 functions as an oncogene in the growth and differentiation of breast cancer cells and hepatoma cancer cells [6, 7]. There is evidence showing that Id4 promoter is hypermethylated in about 30% of primary gastric cancers, and Id4 expression is downregulated in most gastric cancer cell lines due to the hypermethylation in its promoter region [8]. Although Id4 plays different roles in pathogenesis of various cancers, little is known about the functions of Id4 and its regulatory effects on the progression of CRC.

This study aimed to investigate the effects of Id4 on the proliferation, invasion, and metastasis of CRC cells and explore the potential mechanisms.

## 2. Materials and Methods

### 2.1. Cell Lines

The normal colon mucosal cells (NCM460) and CRC HCT116 cells were maintained in McCoy's 5A medium (Invitrogen, CA, USA) at 37°C in 5% CO_2_, the CRC SW480 cells and SW620 cells were maintained in L15 medium (Invitrogen, CA, USA) at 37°C without CO_2_, and the CRC HCT8 cells were grown in RPMI1640 medium (Invitrogen, CA, USA) at 37°C in a humidified environment with 5% CO_2_. The medium was supplemented with 10% fetal bovine serum (FBS).

### 2.2. Plasmid Transfection and Generation of Stably Transduced Cell Lines

Id4 overexpressing and empty lentiviral vectors were provided by the Genechem Biotechnology (Shanghai, China). The stable Id4 overexpressing HCT116 cells (HCT116-Id4) were screened with puromycin at 1.5 *μ*g/ml (Sigma) for at least one week. The Id4 expression was confirmed by quantitative PCR and Western blotting. The primer of Id4 gene was as follows: forward 5'TCGTAAAACCCAGAGCGACC 3', reverse 5' GCTGACTTTCTTGTTGGGCG 3'. pcDNA3.3-CK18 was constructed by inserting a CK18 gene that was generated from PCR product to the BamH1 and Xho I sites (reagents were from New England BioLabs, Beverly, MA, USA) of the plasmid pcDNA3.3-CS2.0-N-flag (Invitrogen). The primers of CK18 gene were as follows: forward, 5'-TAGAGAATTCGGATCCATGAGCTTCACCACTCGCTC-3', reverse, 5'-GCTTCCATGGCTCGAGTTAATGCCTCAGAACTTTGGTG-3'. HCT116 cells were transfected with pcDNA3.3-CK18 or empty vector pcDNA3.3 with Xtreme GENE HP DNA Transfection Reagent (Roche Diagnostics) according to the manufacturer's instructions.

### 2.3. Cell Proliferation Assay

Cell proliferation was assessed by using the WST method. The Id4 overexpressing or empty vector-transfected cells were seeded into 96-well plates at 5 × 10^3^ cells/well and then maintained for 24, 48, or 72 h at 37°C in a humidified environment with 5% CO_2_. Then, 10 *μ*l of WST solution was added to each well, followed by incubation at 37°C for 2 h. The absorbance of each well was determined at 450 nm in a microplate reader.

### 2.4. Colony Formation Assay

In brief, 8 × 10^2^ HCT116-Id4 cells or control HCT116 cells were seeded in 60 mm plates, followed by incubation at 37°C in an environment with 5% CO_2_ for 5–21 days. Surviving colonies containing 50 or more cells were stained with crystal violet, counted, and then photographed under a QImaging MicroPublisher 5.0 RTV microscope.

### 2.5. Cell Cycle Analysis

Cell cycle analysis was performed to measure the DNA content in different phases by flow cytometry (FCM). In brief, cells were harvested and then washed with phosphate-buffered saline (PBS) by centrifugation at 1000×g for 5 min at room temperature. The cells were stained with cell cycle analysis kit at 37°C for 30 min in dark after fixation with 70% ethanol at 4°C, washed with PBS, and then subjected to FCM. The experiments were performed in triplicate.

### 2.6. Wound Healing Assay and Cell Migration/Invasion Assay

HCT116-Id4 cells or control HCT116 cells were seeded in 60 mm plates and grew overnight until about 90% confluence was observed. On the following day, a wound was made in the cell monolayer by scratching. Cells were observed under a light microscope at 0 and 48 h to determine the wound healing of the cell monolayer.

For migration assay, 5 × 10^4^ HCT116-Id4 cells or HCT116 cells in the serum-free medium were seeded into the upper chamber of a Transwell insert (8 mm pore size, BD Bioscience). For invasion assay, the insert was precoated with Matrigel (BD Bioscience), and 8 × 10^5^ cells were seeded into the insert. The medium containing 20% FBS was added into the lower chamber as a chemoattractant. Cells that remained on the upper chamber after incubation for 48 h were removed by a cotton swab, and the insert was subsequently stained with 0.1% crystal violet in 20% methanol, followed by cell counting after photographing under a QImaging MicroPublisher 5.0 RTV microscope.

### 2.7. Western Blotting

After washing with cold PBS, HCT116 cells in 6 cm dishes were lysed with cell lysis buffer (50 mM HEPES, pH 7.4, 250 mM NaCl, 1% Nonidet P-40, 1 mM EDTA, 1 mM Na_3_VO_4_, 1 mM NaF, 1 mM PMSF, and 1 mM dithiothreitol and protease inhibitor cocktail from Roche) for 30 min at 4°C for Western blotting or coimmunoprecipitation (IP). The protein concentration was determined by using the BCA protein Assay Kit (Pierce, USA), and then, proteins of the same amount were separated by 12% or 15% SDS-PAGE. Then, proteins were transferred onto PVDF membranes (GE Healthcare), which were blocked in Tris-buffered saline containing 5% bovine serum albumin (BSA) for 1 h at room temperature. The primary antibodies used in this study included anti-Id4 (1 : 200; Santa Cruz Biotechnology, Santa Cruz, CA, USA), anti-P21 (1 : 1000, Cell Signaling Technology, USA), anti-P27 (1 : 1000, Cell Signaling Technology, USA), anti-PI3K (1 : 1000, Cell Signaling Technology, USA), anti-P-PI3K (1 : 1000, Cell Signaling Technology, USA), anti-AKT (1 : 1000, Cell Signaling Technology, USA), anti-P-AKT (1 : 1000, Cell Signaling Technology, USA), anti-GAPDH (1 : 1000, Cell Signaling Technology, USA), anti-MMP2 (1 : 5000, Abcam, USA), anti-MMP9 (1 : 2000, Abcam, USA), anti-MMP7 (1 : 5000, Abcam, USA), anti-Twist (1 : 1000, Cell Signaling Technology, USA), anti-slug (1 : 1000, Cell Signaling Technology, USA), anti-*β*-catenin (1 : 1000, Cell Signaling Technology, USA), anti-snail (1 : 1000, Cell Signaling Technology, USA), anti-TIMP1 (1 : 1000, Cell Signaling Technology, USA), anti-TIMP2 (1 : 1000, Cell Signaling Technology, USA), and anti-CK18 (1 : 3000, Abcam, USA). After incubation with horseradish peroxidase-conjugated secondary antibodies, the membranes were visualized by using the enhanced chemiluminescence method (ECL Plus, USA).

### 2.8. Coimmunoprecipitation (Co-IP) Assay

For the Co-IP assay, the HCT16-Id4 cells were lysed, and the soluble proteins were precleared with 100 *μ*l of 50% slurry of protein A agarose (Invitrogen). The clear lysates were then mixed with 4 *μ*g of antibodies and 100 *μ*l of 50% slurry of protein A agarose. The immunoprecipitated complexes were analyzed by Western blotting.

### 2.9. Animal Experiments

Specific pathogen-free (SPF) BALB/C nude mice aged 4–6 weeks were purchased from Shanghai SLAC Laboratory Animal Co. Ltd. All experiments were performed in accordance with Animal Care Committee of Fujian Medical University, China. For in vivo tumor growth assessment, 1 × 10^6^ HCT116-con cells and HCT116-Id4 cells in 100 *μ*l of PBS were subcutaneously injected into the left and right flank of mice, respectively. The tumor volume was measured once every 4 days and calculated as follows: (length × width)^2^/2. Two weeks later, the tumor weight was obtained after the mice were sacrificed.

For the establishment of the liver metastasis model, 5 × 10^6^ cells were injected into the spleen of each mouse. At days 28, the mice were sacrificed, and the tumor samples were collected from the site of the tumor injection and from the liver and calculated. All the tissues were fixed in 4% formalin and then embedded with paraffin for further pathological examination.

### 2.10. Statistical Analysis

Data are expressed as means ± standard deviation (SD) from at least 3 independent examinations, and statistical analysis was performed with Prism7 (GraphPad Software, La Jolla, CA, USA). Comparisons were performed with Student's *t*-test between groups or one-way analysis of variance (ANOVA) among groups. A value of *P* < 0.05 was considered statistically significant.

## 3. Results

### 3.1. Id4 Overexpression Inhibits the Proliferation of HCT116 Cells

**Figure 1:**
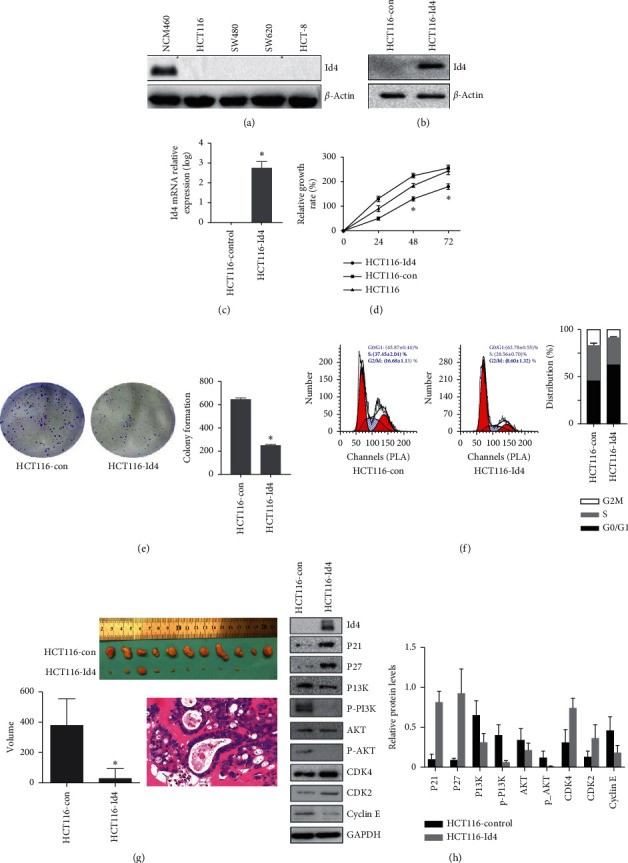
Id4 overexpression reduces cell proliferation and arrests cell cycle at the G0/G1 phase. (a) Id4 protein expression in the CRC. (b, c) Real-time RT-PCR and Western blotting confirmed stable Id4 overexpression in the HCT116 cells. *β*-Actin was used as a loading control. (d) WST assay was used to assess the effect of Id4 on the proliferation of HCT116 cells. (e) Colony formation assay was used to assess the effect of Id4 on the colony-forming ability of the HCT116 cells. (f) Flow cytometry was performed to detect the cell cycle after Id4 overexpression. (g) Photographs of the xenograft tumors in the nude mice at day 14 after implantation, and tumor weight was measured. (h) Protein expression of molecules in the PI3K/AKT pathway and proliferation-related proteins in the HCT116-Id4 cells (Western blotting). GAPDH was used as a loading control. Data are expressed as mean ± SD. ^*∗*^*P* < 0.05.

The Id4 expression was detected in the four CRC cell lines (HCT116, SW480, SW620, and HCT8) and the normal colon mucosal cells (NCM460). Results showed the Id4 protein expression reduced significantly in these 4 CRC cell lines as compared to the NCM460 cells (Figure 1(a)).

To examine the effects of Id4 on the biological characteristics of CRC, HCT116 cells with stable Id4 expression (HCT116-Id4 cells) were established, and the Id4 expression was confirmed by Western blotting and real-time RT-PCR (Figures 1(b) and 1(c), *P* < 0.05). Subsequently, the cell proliferation of HCT116-Id4 cells and control cells was determined. As shown in Figure 1(d), the proliferation of the HCT116-Id4 cells was significantly inhibited as compared to the control group (*P* < 0.05), indicating that Id4 might play a key role in the growth of HCT116 cells. The colony-forming assay confirmed the inhibitory effect of Id4 on the proliferation of the HCT116 cells (Figure 1(e)). To investigate if Id4 is involved in cell cycle progression of HCT116 cells, FCM was performed to detect the percentage of cells in different cell phases. Results showed that the proportion of HCT116 cells in the G1/G0 phase increased significantly following Id4 transfection (*P* < 0.01), but the proportions of HCT116 cells in the S phase and G2/M phase markedly decreased (*P* < 0.01) (Figure 1(f)). These results demonstrated that Id4 might regulate cell cycle progression of HCT116 cells. Moreover, markedly smaller tumors were found in nude mice after HCT116-Id4 cells inoculation as compared to the control group (Figure 1(g), *P* < 0.05), which illustrated that Id4 could significantly inhibit the growth of xenograft tumor in the nude mice. Collectively, these findings suggested that Id4 might be a novel tumor suppressor in the CRC.

Furthermore, to investigate the underlying mechanisms underlying the regulatory effects of Id4 on the CRC cells, the expression of proliferation-related markers was detected in the HCT116-Id4 cells. Results showed that upregulation of Id4 expression markedly increased the expression of P21, P27, CDK4, and CDK2 but significantly decreased the expression of PI3K, P-PI3K, AKT, p-AKT (upstream molecules of P21 and P27), and cyclin E in the HCT116-Id4 cells as compared to the control HCT116 cells (Figure 1(h), *P* < 0.05). These results demonstrated that Id4 upregulated the expression of proliferation-related proteins and inhibited the PI3K/AKT pathway in the CRC cells.

### 3.2. Id4 Inhibits the Migration, Invasion, and Metastasis of CRC Cells In Vitro and In Vivo

**Figure 2:**
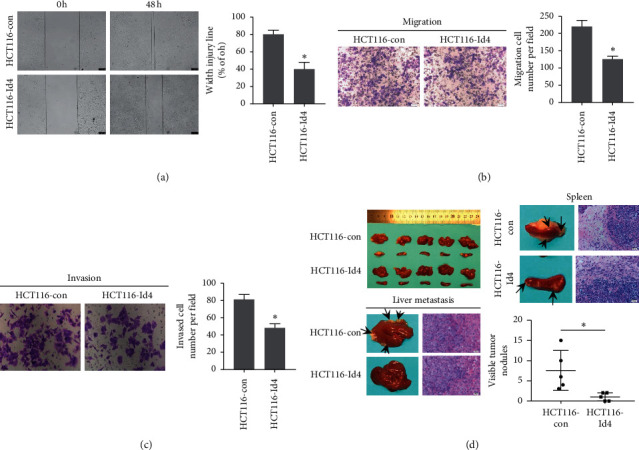
Id4 regulated the migration, invasion, and metastatic potentials of CRC cells. (a) Wound healing assay of CRC cells. (b) Migration assay of CRC cells (Boyden chamber assay). (c) Invasion assay of CRC cells (Boyden chamber assay). (d) Intrasplenic implantation of HCT116-Id4 cells or control cells. The number of tumors in the spleens and liver metastases of the HCT116-Id4 group decreased significantly.

In order to explore the effects of Id4 on the migration of CRC cells, a wound healing/scratch assay was performed. As shown in Figure 2(a), Id4 upregulation remarkably attenuated the wound healing as compared to the control HCT 116 cells. The invasive potential of HCT116-Id4 cells and control cells was assessed by a modified Boyden chamber invasion assay. Results showed Id4 overexpression significantly reduced the HCT116-Id4 cells invading the Matrigel layer as compared to the control HCT 116 cells (*P* < 0.01, Figures 2(b) and Figure 2(c)). To assess the metastatic ability of HCT116-Id4 cells in vivo, 2 × 10^6^ cells were injected into the spleens of nude mice. Four weeks later, all the mice in the control group developed liver metastases (5/5). In contrast, only 1 mouse (1/5) in the HCT116-Id4 group developed liver metastases (*P* < 0.05). The number of nodules formed in the liver of the mice implanted with HCT116-Id4 cells remarkably reduced as compared to mice implanted with the control cells (Figure 2(d)). Collectively, these results suggested an important role of Id4 on the progression of CRC.

### 3.3. Id4 Suppresses the Epithelial-Mesenchymal Transition of HCT116 Cells

**Figure 3:**
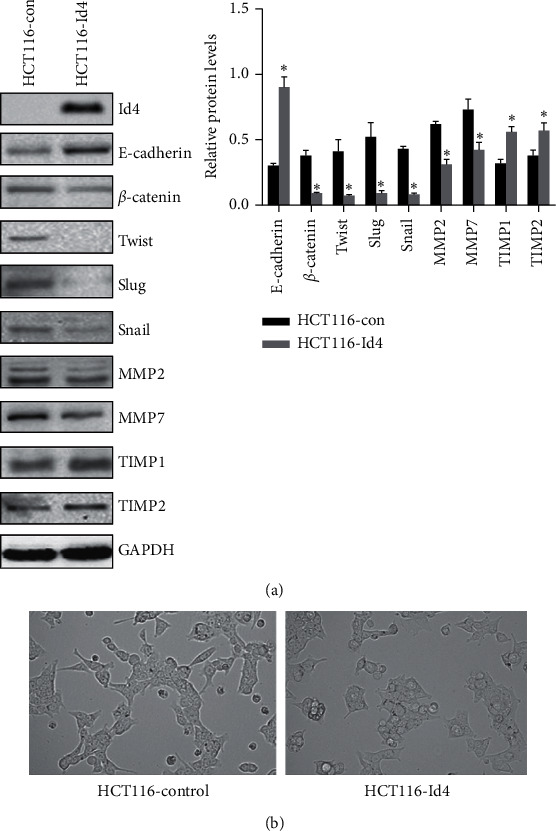
Id4 regulated the expression of EMT-related markers in the HCT116 cells. (a) The expression of EMT-related molecules in the HCT116-Id4 cells and control cells (Western blotting). (b) Cellular morphology of HCT116-Id4 cells and control cells under microscopy.

Epithelial-mesenchymal transition (EMT) plays a crucial role in the metastasis of many cancers. To investigate the phenotypic changes of HCT116 cells after Id4 overexpression, the expression of EMT-associated molecules was detected in the HCT116 cells by Western blotting. At the same time, the expression of MMP2, MMP7, and MMP9 (factors in the matrix metalloproteinases family and involved in the invasion of cancers) was detected in the control cells and HCT116-Id4 cells. Western blotting revealed that the protein expression of *β*-catenin, twist, slug, snail, MMP2, MMP7, and MMP9 significantly decreased, while the expression of E-cadherin, TIMP1, and TIMP2 was markedly upregulated in the HCT116-Id4 cells as compared to control cells (Figure 3(a), *P* < 0.05). Microscopy showed that Id4 overexpressing cells acquired more characteristics of epithelial morphology than the control cells (Figure 3(b)). These revealed a possible molecular mechanism about the Id4-mediated cell migration and invasion.

### 3.4. Id4 Regulates CK18 Expression in the CRC and Mediates the Migration and Invasion through CK18

**Figure 4:**
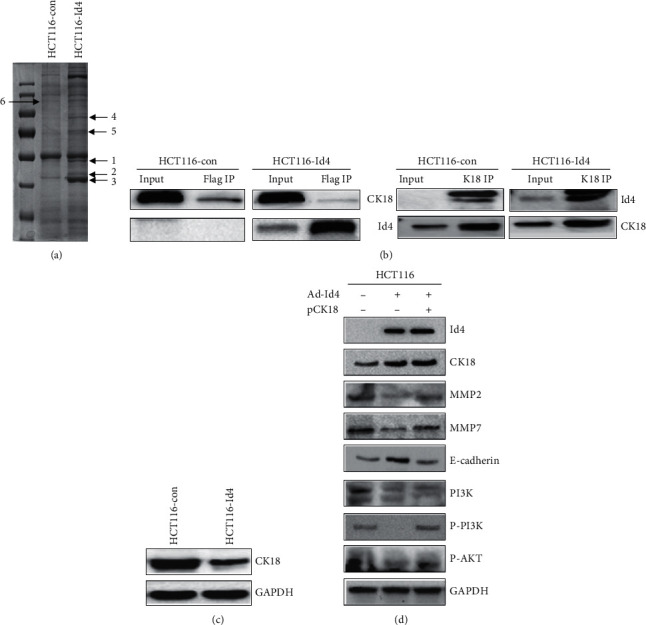
Id4 binding to CK18 regulated the PI3K pathway and the expression of EMT-related markers. (a) Coomassie blue-stained SDS-PAGE gel showed the protein binding to Id4 (flag). (b) Co-IP assay showed the interaction between Id4 and CK18 in the HCT116 cells. (c) Id4 decreased CK18 protein expression (Western blotting). (d) HCT116-Id4 cells and negative control cells were transfected with CK18 plasmid or control plasmid, respectively. Western blotting was performed to investigate the effect of CK18 on the expression of EMT-related markers in the HCT116 cells.

To explore the downstream effectors of Id4 in the HCT116 cells, proteins which were likely to interact with Id4 were captured by immunoprecipitation assay, and then, the proteins were separated by using the SDS-PAGE method and identified by MALDI-TOF-MS (Figure 4(a)). Seven differentially expressed proteins were identified in the HCT116-Id4 cells as compared to control cells. Of which, the cytokeratin 18 (CK18) was selected for further confirmation. An in vivo coimmunoprecipitation (Co-IP) assay was performed in the HCT116-Id4 cells and HCT116 cells to investigate the potential interaction between Id4 and CK18 in the CRC cells. As shown in Figure 4(b), Id4 was able to efficiently coprecipitate with endogenous CK18 and vice versa. These results revealed that Id4 could interact with CK18 in vivo.

Subsequently, to assess the functional consequence of the interaction between Id4 and CK18, the following experiments were performed to explore whether Id4 could affect the CK18 protein expression. As shown in Figure 4(c), Id4 overexpression significantly decreased the CK18 protein expression in the HCT116-Id4 cells. Thus, we hypothesized that Id4 attenuates the motility, invasion, and migration of CRC cells through CK18. To further validate this hypothesis, Id4 was compensated by its exogenous protein in the HCT116-Id4 cells as a genetic rescue experiment. As shown in Figure 4(d), CK18 expression in the HCT116-Id4 cell was restored to the control level after transfection with the CK18 overexpressing vector. Consequently, HCT116-Id4/CK18 overexpressing cells showed the upregulated expression of MMP2, MMP7, PI3K, P-PI3K, and p-AKT, but the E-cadherin expression was downregulated as compared to the HCT116-Id4 cells (Figure 4(d)). Taken together, our findings suggested Id4 affects the CK18 expression and the PI3K/AKT pathway via CK18.

## 4. Discussion

The Id family has helix loop-helix transcription factors and is one of the key regulators of cell fate, differentiation, proliferation, and cell death [9, 10]. Several reports have revealed the importance of Id proteins in the inhibition of cell differentiation and regulation of cell proliferation. In many types of human tumors, the Id protein expression increases which is often associated with the enhanced malignancy and aggressive clinical behaviours [11, 12]. In our previous study, results showed Id1 expression increased significantly in the CRC tissues than in the normal mucosal tissues, and Id1 expression was positively related to poor differentiation of CRC cells [13]. Moreover, knock-down of Id1 gene not only inhibits the growth of HCT116 cells but also suppresses the hepatic metastasis in vivo in a CXCR4-dependent manner [14]. These results underscore the importance of Id-mediated tumorigenesis, cell proliferation, and metastasis of cancers.

Despite the structural similarity, Id4 still has different roles in the tumorigenesis, tumor invasion, and metastasis as compared to other Id family members. Although the Id4 protein is involved in the regulation of cell cycle and cell senescence [15], its expression and functions remain controversial in some cancers. Zeng et al. [16] found that Id4 was highly expressed in the glioblastoma multiforme (GBM), in which Id4 promoted the growth and angiogenesis. However, in the prostate cancer, Id4 acted as a tumor suppressor, and Id4 overexpression in the PC3 cells (one of the highly malignant prostate cancer cell lines) increased apoptosis and inhibited cell proliferation and migration [5, 17]. Id4 has also been found to be epigenetic silencing in many cancers such as squamous cell carcinoma [18], gastric cancer [8], and CRC [19], which is ascribed to the promoter hypermethylation. Id4 gene promoter is hypermethylated in 30% of primary gastric cancers, and Id4 expression is downregulated in most gastric cancer cell lines due to the hypermethylation of the promoter region [8]. Although the methylation state of Id4 gene promoter in the CRC still needs to be investigated thoroughly, Id4 might be a tumor suppressor in the CRC according to our findings that the Id4 expression was very low in the CRC cells than in the normal mucosal cells.

The effects of Id4 on the cell cycle and proliferation have been reported in some cancer cells. Id4 overexpression is able to arrest cell cycle and inhibit cell proliferation, which is associated with the increased expression of cyclin-dependent kinase inhibitors p21 and p27 in the prostate cancer cell line DU145 cells [5]. In addition, Id4 overexpression in lung cancer cells inhibits cisplatin-induced apoptosis via the p38 MAPK pathway [20]. Similar findings were observed in our study. Id4 overexpression significantly reduced tumor growth and hepatic metastasis in the mice inoculated with HCT116-Id4 cells. The cell cycle was arrested at the G0/G1 phase in the HCT116-Id4 cells, which was further confirmed by the increased expression of p21 and p27. In addition, Id4 overexpression suppressed the expression and activity of PI3K, p-PI3K, AKT, and p-AKT, suggesting that Id4 induces the suppression of CRC cell growth which may be related to the inhibition of the PI3K/AKT signaling pathway. EMT is a process which involves in the shifting between two alternative states, mesenchymal or epithelial. Aberrant EMT activation may trigger the dissociation of cancer cells from primary carcinomas, and then, they subsequently migrate and disseminate to distant organs. During the EMT, the expression of cell adhesion molecules, especially E-cadherin, is concomitantly repressed, but that of other molecules (such as snail, zeb, and twist) is upregulated [21]. Numerous studies have indicated that Id protein is one of factors involved in the EMT. In lung cancer cells, the inhibition of Id1 expression reduces the expression of EMT-related markers (vimentin, intergrin-*β*, and snail). Even in the cancer tissues from NSCLS patients, Id1 seems to be significantly related to the vimentin and other EMT-related proteins [22]. Id1 opposed twist activity, thus promoting cell metastasis to the lung via EMT [23]. It has been indicated that Id4 can inhibit lung cancer metastasis and mesenchymal-epithelial transition (MET) by binding to slug and promoting E-cadherin expression [24]. MMPs are also important markers of EMT and play a major role in the biological behaviours of cells such as cell proliferation, migration, and differentiation. MMP2 and MMP9 are gelatinases capable of degrading type IV collagen and are the most abundant components in the basement membrane. The degradation of the basement membrane is an essential step for the metastasis of most cancers [25]. MMP7 is also known as matrilysin which can digest components of the extracellular matrix. The active MMP7 can also cleave the pro-MMP2 and pro-MMP9 to facilitate tumor invasion [26]. Id4 is involved in suppressing the MMP2-mediated cell invasion in glioblastoma [27]. Our results showed that Id4 overexpression downregulated the expression of MMP2, MMP7, *β*-catenin, twist, slug, and snail, but upregulated that of TIMP1 and TIMP2, indicating that Id4 is involved in reversing the progression of EMT.

Id proteins dimerize with bHLH protein, which inhibits their DNA binding activity [28]. In the lung cancer, Id4 binds to slug, which interferes with its interaction with E-box promoter, and then suppresses the metastasis of cancer cells [24]. Id4 interacts with MDC1 and other DNA repair proteins which govern the DNA damage response [29]. Our results showed that Id4 could interact with CK18. CK18 gene encodes the type I intermediated filament protein, which is primarily found in many types of single-layered epithelial tissues. CK18 protein localizes in the cytoplasm and perinuclear region [30]. CK18 is important for some cell processes including apoptosis [31], mitosis [32], cell cycle progression [33], and cell signaling [34]. Prior studies have noted that CK18 is an epithelial marker in the diagnosis of cancers. Galarneau et al. [34] proposed that CK18 was involved in the signalling pathways related to the regulation of cell growth, death, and motility. The PI3K/AKT pathway plays a pivotal role in these mechanisms in which CK18 involves. There is a close relationship between CK18 and AKT because AKT1 overexpression increases the CK8/18 expression and AKT2 upregulates the expression of CK18 and vimentin [35]. According to our findings, CK18 overexpression reverses the suppressive effects of Id4 on the PI3K/AKT pathway and EMT. It means that CK18 may be a target of Id4 in the PI3K/AKT pathway. Together, findings in the present study indicate Id4 can inhibit the growth and invasion of CRC cells through CK18-related inhibition of the AKT pathway and EMT.

## 5. Conclusion

In summary, our findings indicate that Id4 expression reduces in the CRC cells, and Id4 overexpression inhibits the growth of CRC cells as well as suppresses the migration, invasion, and metastasis of CRC cells in vitro and in vivo. Furthermore, Id4 mediates the migration and invasion of CRC cells through interacting with CK18 to suppress the PI3K/AKT pathway and EMT. Collectively, these findings implicate that Id4 may serve as a tumor suppressor and has the promise to become a target in the clinical treatments of CRC.

## Data Availability

The data used to support the findings of this study are available from the corresponding author upon request.
